# Measuring change in trials of physical activity interventions: a comparison of self-report questionnaire and accelerometry within the PACE-UP trial

**DOI:** 10.1186/s12966-018-0762-5

**Published:** 2019-01-22

**Authors:** Elizabeth S. Limb, Shaleen Ahmad, Derek G. Cook, Sally M. Kerry, Ulf Ekelund, Peter H. Whincup, Christina R. Victor, Steve Iliffe, Michael Ussher, Julia Fox-Rushby, Cheryl Furness, Judith Ibison, Stephen DeWilde, Tess Harris

**Affiliations:** 1 Population Health Research Institute, St George’s University of London, Cranmer Terrace, Tooting, London, SW17 ORE UK; 2 Pragmatic Clinical Trials Unit, Queen Mary’s University of London, London, E1 2AT UK; 3 Department of Sport Medicine, Norwegian School of Sport Sciences, PO Box 4014, 0806 Oslo, Norway; 4 Gerontology and Health Services Research Unit, Brunel University, London, UB8 3PH UK; 5 Research Department of Primary Care & Population Health, University College, London, NW3 2PF UK; 6 Present address: Department of Population Health Sciences, King’s College London, London, SE1 1UL UK

**Keywords:** Walking, Intervention, Primary care, MVPA, Accelerometry, IPAQ, GPPAQ

## Abstract

**Background:**

Few trials have compared estimates of change in physical activity (PA) levels using self-reported and objective PA measures when evaluating trial outcomes. The PACE-UP trial offered the opportunity to assess this, using the self-administered International Physical Activity Questionnaire (IPAQ) and waist-worn accelerometry.

**Methods:**

The PACE-UP trial (*N* = 1023) compared usual care (*n* = 338) with two pedometer-based walking interventions, by post (*n* = 339) or with nurse support (*n* = 346). Participants wore an accelerometer at baseline and 12 months and completed IPAQ for the same 7-day periods. Main outcomes were weekly minutes, all in ≥10 min bouts as per UK PA guidelines of: i) accelerometer moderate-to-vigorous PA (Acc-MVPA) ii) IPAQ moderate+vigorous PA (IPAQ-MVPA) and iii) IPAQ walking (IPAQ-Walk). For each outcome, 12 month values were regressed on baseline to estimate change.

**Results:**

Analyses were restricted to 655 (64%) participants who provided data on all outcomes at baseline and 12 months. Both intervention groups significantly increased their accelerometry MVPA minutes/week compared with control: postal group 42 (95% CI 22, 61), nurse group 43 (95% CI 24, 63). IPAQ-Walk minutes/week also increased: postal 57 (95% CI 2, 112), nurse 43 (95% CI -11, 97) but IPAQ-MVPA minutes/week showed non-significant decreases: postal -11 (95% CI -65, 42), nurse -34 (95% CI -87, 19).

**Conclusions:**

Our results demonstrate the necessity of using a questionnaire focussing on the activities being altered, as with IPAQ-Walk questions. Even then, the change in PA was estimated with far less precision than with accelerometry. Accelerometry is preferred to self-report measurement, minimising bias and improving precision when assessing effects of a walking intervention.

Trial registration: ISRCTN, ISRCTN98538934. Registered 2 March 2012.

## Background

Adults who participate in regular physical activity (PA) and remain fit and active into later life have fewer chronic health conditions, and are better able to maintain a healthy weight [1]. WHO, UK and US aerobic PA guidelines for adults recommend at least 150 min weekly of moderate-to-vigorous-physical-activity (MVPA) in bouts of at least 10 min, or 75 min of vigorous PA, or a combination. Brisk walking (3 miles/hr. or 5 km/hr) counts as MVPA [2] and for most people approximates to 1000 steps in 10 min [3].

Self-report questionnaires are a quick, easy way to assess PA. Population surveys such as the Health Survey for England (HSE) [4] and Sport England’s “Active Lives Survey” [5] use self-completed questionnaires and report estimates that around 60% of participants aged 16+ meet PA guidelines. However, individuals often over-estimate their PA, particularly walking, on questionnaires compared with accelerometry measures of MVPA [6–8]. Self-report questionnaires can thus lead to inflated estimates of “active” individuals [9].

The International Physical Activity Questionnaire (IPAQ) short form [10] assesses 7-day recall of PA in ≥10 min bouts based on intensity (separating vigorous, moderate and walking activity) and duration (days per week and minutes per day). The shorter General Practice Physical Activity Questionnaire (GPPAQ) [11] does not provide a continuous measure of PA, but categorises individuals as active or not. GPPAQ is used in the UK National Health Service (NHS) primary care cardiovascular health checks [12]. Individuals classified as less than “active” are assumed not to be meeting PA guidelines and are offered advice. In contrast, accelerometry is an objective PA measure, providing information on step-counts and time spent in different PA intensities and is increasingly being used in cross-sectional studies to study PA [13, 14]. Although accelerometers e.g. Actigraph are not a gold standard for measuring PA, they have been shown to correlate well with doubly labelled water to measure activity energy expenditure [15]. For the Actigraph GT3X accelerometer, standard cut-points for accelerometer counts per minute (CPM) for different PA intensity categories have also been defined, thus leading to assessment of time spent in different PA intensities: light 101–1951 CPM; moderate 1952–5724 CPM; vigorous ≥5725 CPM [16].

Longitudinal studies and trials which examine PA changes over time need valid, reliable PA assessment methods. Both IPAQ-Short and accelerometry have been used separately to measure PA change over time, [17–20] but only a few small studies have used both and compared change in minutes of PA [21, 22]. Other studies have compared self-report PA minutes with either pedometer steps [23, 24] or accelerometry counts [25] which are not directly comparable. The PACE-UP trial offers the opportunity to directly compare change in PA minutes from accelerometry and IPAQ within a large trial dataset. This study had the following objectives: to compare the trial treatment effects at 12 months (difference between intervention and control groups in the change in PA) using (i) accelerometry minutes of MVPA and IPAQ minutes of moderate+vigorous activity and walking; (ii) the percentage of “active” individuals classified by accelerometry, IPAQ and GPPAQ.

## Methods

### Background to the PACE-UP study

The PACE-UP study is a three-arm parallel groups randomised controlled trial comparing a 3-month pedometer-based walking intervention, delivered by post or with nurse support, to usual care [26]. Ethical approval was given by the London Research Ethics Committee (Hampstead) (12 L/LO/0219), trial registration ISRCTN 98538934. Adults aged 45–75 years from seven South-West London (UK) General Practices (family practices) who self-reported as inactive were invited to take part. Following a baseline assessment to assess eligibility, 1023 participants gave informed written consent and were randomised into one of three groups: the Control group (*n* = 338) received usual care; the Postal group (*n* = 339) received a pedometer, a 12-week personalised walking plan including behaviour change techniques (e.g. goal setting, self-monitoring) designed to increase their walking and a step-count diary through the post; the Nurse group (*n* = 346) received these and were additionally offered three individual practice nurse PA consultations. Randomisation was carried out at household level allowing couples to take part together. The main trial outcomes were changes in accelerometry measured average daily step-count and total weekly time in MVPA in ≥10 min bouts between baseline and 12 months. 956/1023 (93%) provided at least 1 day of accelerometry data at 12 months, > 90% provided at least 5 days wear. The postal and nurse groups both significantly increased their objective PA levels (step count and time in MVPA) compared with the control group, with no difference between intervention groups at 12 months [27].

Participants wore a sealed accelerometer (GT3X, Actigraph LLC) over their hip for 7 consecutive days at baseline, prior to randomization, and 12 months post-randomization. They also completed the IPAQ Short form [10] and GPPAQ [11], both designed for self-completion, for the same 7-day periods as they wore the accelerometer. Actilife software (v 6.6.0) was used to extract and reduce the Actigraph data, ignoring runs of ≥60 min of zero counts [26], to provide daily steps counts and time spent in ≥10 min bouts of MVPA (≥1952 counts per minute, equivalent to ≥3 Metabolic Equivalents (METs)) [16]. When assessing ≥10 min bout, the default “drop time” of 2 min was used, which allows for a 2 min interruption in bout activity. At baseline, all participants provided ≥5 days of ≥540 min accelerometer wear-time. To limit attrition bias, those providing ≥1 day of ≥540 min accelerometer wear time at 12 months were included in analyses. IPAQ questions focus on time spent being physically active in the previous 7 days in at least 10 min bouts, including PA at work, home, travelling and leisure. For each of vigorous and moderate PA and walking, there are questions on the number of days and the duration on each of these days. GPPAQ questions ask about PA at work and the type and weekly duration of leisure PA (physical exercise/sport, cycling, walking, housework/childcare and gardening/DIY). Duration categories are None, < 1 h, 1–3 h, ≥3 h.

### Study outcomes

#### Accelerometry

The main accelerometry outcome was total weekly minutes of MVPA in ≥10 min bouts; a secondary outcome was total weekly minutes of MVPA, including MVPA in < 10 min bouts. Binary variables were generated for each MVPA outcome to indicate 150 min of activity.

#### IPAQ

Total weekly minutes spent in each of vigorous PA, moderate PA and walking were calculated, capped at a maximum of 3 h/day or 21 h/week, as recommended by the IPAQ coding guidelines [28]. Two self-report PA measures were derived: total weekly minutes of vigorous + moderate PA in bouts of ≥10 min, excluding walking (IPAQ-MVPA) and total weekly minutes of walking in bouts of ≥10 min (IPAQ-Walk). We also report an additional outcome, IPAQ-Total (IPAQ-MVPA + IPAQ-Walk), conceptually the same construct as accelerometry MVPA in ≥10 min bouts. Binary variables were generated for each of these to indicate 150 min or more per week of activity.

#### GPPAQ

The GPPAQ Physical Activity Index is a 4-level index ranging from “Inactive” through to “Active”. “Active” individuals are achieving ≥3 h (180 min) of MVPA per week including work PA and leisure PA from physical exercise and cycling, but not including PA from walking, housework/childcare or gardening. We defined a binary outcome, GPPAQ, to identify those individuals classified as “Active” by the GPPAQ score. However, adults who are retired or not working and who do no sport or cycling can never be classified as active, although they may achieve MVPA guidelines through walking. Thus, a modified index, GPPAQ-Walk, was also derived, where those who reported walking briskly for at least 3 h per week were classified as “active”. Previous analysis of GPPAQ showed this modified index had improved sensitivity at identifying active individuals compared with accelerometry data, but lower specificity in adults aged 60–75 years [29].

### Statistical analyses

Analyses were carried out using Stata 14 [30]. Multi-level regression models estimated treatment effects for accelerometer, IPAQ and GPPAQ outcomes. The 12-month outcome was regressed on baseline value, treatment group, age, gender, practice and month of baseline accelerometry as fixed effects and household as a random effect in the multi-level model. (i) Linear regression was used for weekly minutes of accelerometer MVPA, IPAQ-MVPA, IPAQ-Walk and IPAQ-Total; (ii) logistic regression was used for the binary variables “active” from accelerometry, IPAQ and GPPAQ. The distributions of change in PA for the four continuous outcomes were reasonably normally distributed, as were the distributions of residuals from the models, allowing this method of analysis. Analyses were restricted to those with complete data for all outcomes being compared: 833 at baseline and 655 for the longitudinal regression models. This ensured direct comparisons of the same group of participants for each outcome. Sensitivity analyses used ≥180 min of accelerometer MVPA and IPAQ outcomes, as the GPPAQ outcome is based on ≥180 min per week.

## Results

At baseline, accelerometry data were available on all participants and 989 (97%) returned IPAQ and GPPAQ questionnaires. At 12 months, 956 (93%) participants provided at least 1 day of accelerometry and 942 (92%) returned IPAQ and GPPAQ questionnaires. However, incomplete answers on IPAQ and GPPAQ questions reduced the sample size to 833 at baseline and to 655 for analyses of changes between baseline and 12 months. Study groups were balanced at baseline for the 833 with complete data with respect to age, gender, ethnicity and different health measures (Table 1). One third of participants were male and two thirds were overweight or obese (Body Mass Index ≥25 kg/m^2^). Mean weekly minutes of accelerometer-MVPA were 317 (sd 151) for total MVPA and 98 (sd 103) for MVPA in ≥10 min bouts. Self-reported mean weekly minutes were 174 (sd 279) for IPAQ-MVPA, 315 (sd 310) for IPAQ-Walk, similar to total accelerometry MVPA and 489 (sd 453) for IPAQ-Total. Accelerometry data classified 23% of participants at baseline as “Active” i.e. achieving ≥150 min of MVPA per week in ≥10 min bouts (Table 1). In contrast, 35, 66 and 84% of participants self-reported ≥150 min per week of IPAQ-MVPA, IPAQ-Walk and IPAQ-Total respectively. GPPAQ classified 12% of participants as active which increased to 28% when walking was included.

**Table 1:** Demographic, health, physical characteristics and physical activity at baseline

	All groups (*N* = 833)	Control (*N* = 279)	Postal (*N* = 270)	Nurse (*N* = 284)
	n (%)	n (%)	n (%)	n (%)
Age at randomisation				
45–54 years	280 (34%)	87 (31%)	94 (35%)	99 (35%)
55–64 years	315 (38%)	111 (40%)	98 (36%)	106 (37%)
65–75 years	238 (29%)	81 (29%)	78 (29%)	79 (28%)
Sex: Male	304 (36%)	98 (35%)	104 (39%)	102 (36%)
Ethnicity				
White	654 (81%)	212 (79%)	222 (85%)	220 (80%)
Black / African / Caribbean / Black British	77 (10%)	25 (9%)	21 (8%)	31 (11%)
Asian / Asian British	54 (7%)	21 (8%)	14 (5%)	19 (7%)
Other, incl mixed	19 (2%)	10 (4%)	4 (2%)	5 (2%)
General health: Very good or good	679 (83%)	223 (81%)	230 (88%)	226 (82%)
Chronic diseases				
None	321 (39%)	109 (39%)	112 (42%)	100 (36%)
1–2	436 (53%)	153 (55%)	133 (50%)	150 (54%)
≥ 3	61 (7%)	14 (5%)	20 (8%)	27 (10%)
Self-reported pain: Yes	566 (69%)	185 (67%)	191 (72%)	190 (69%)
Limiting long-standing illness	174 (21%)	60 (22%)	55 (21%)	59 (21%)
Townsend Disability score				
None (0)	491 (60%)	159 (58%)	158 (59%)	174 (62%)
Slight or some disability (1–6)	305 (37%)	103 (37%)	104 (39%)	98 (35%)
Appreciable or severe disability (7–18)	24 (3%)	13 (5%)	4 (2%)	7 (3%)
Physical characteristics				
Overweight/obese: BMI ≥ 25 kg/m^2^	544 (65%)	184 (66%)	173 (64%)	187 (66%)
	**Mean (sd)**	**Mean (sd)**	**Mean (sd)**	**Mean (sd)**
Fat mass (kg)	26 (11)	26 (10)	26 (11)	26 (11)
Accelerometry data				
Adjusted baseline step count per day	7550 (2670)	7528 (2685)	7480 (2583)	7638 (2744)
Total weekly mins MVPA in ≥10 min bouts	98 (103)	91 (100)	97 (94)	106 (113)
Total weekly mins MVPA	317 (151)	316 (152)	311 (145)	322 (154)
Daily wear time (mins)	792 (79)	791 (73)	789 (79)	796 (84)
International Physical Activity Questionnaire (IPAQ)				
IPAQ-MVPA: Weekly mins of moderate PA + vigorous PA in ≥10 min bouts	174 (279)	194 (310)	159 (266)	167 (259)
IPAQ-Walk: Weekly mins of walking in ≥10 min bouts	315 (310)	323 (327)	316 (326)	307 (275)
IPAQ-Total: Weekly mins of moderate PA + vigorous PA + walking in ≥10 min bouts	489 (453)	518 (501)	475 (457)	474 (395)
	**n (%)**	**n (%)**	**n (%)**	**n (%)**
Proportions of “active” individuals^a^				
Accelerometry				
150 weekly mins MVPA in ≥10 min bouts	190 (23%)	57 (21%)	58 (22%)	75 (27%)
International Physical Activity Questionnaire (IPAQ)				
150 weekly mins of IPAQ-MVPA	286 (35%)	99 (36%)	86 (32%)	101 (36%)
150 weekly mins of IPAQ-Walk	540 (66%)	176 (64%)	173 (65%)	191 (68%)
150 weekly mins of IPAQ-Total	690 (84%)	227 (82%)	226 (85%)	237 (84%)
General Practice Physical Activity Questionnaire (GPPAQ)				
GPPAQ: “Active” ≥180 mins PA per week	101 (12%)	38 (14%)	33 (12%)	30 (11%)
GPPAQ-Walk: “Active” ≥180 mins PA per week including walking at brisk/fast pace	229 (28%)	82 (30%)	71 (27%)	76 (27%)

^a^Proportions of “active” individuals are based on 276, 265 and 281 participants in Control, Postal and Nurse groups respectively

### i) Comparison of estimated treatment effects using minutes of physical activity

**Fig. 1:**
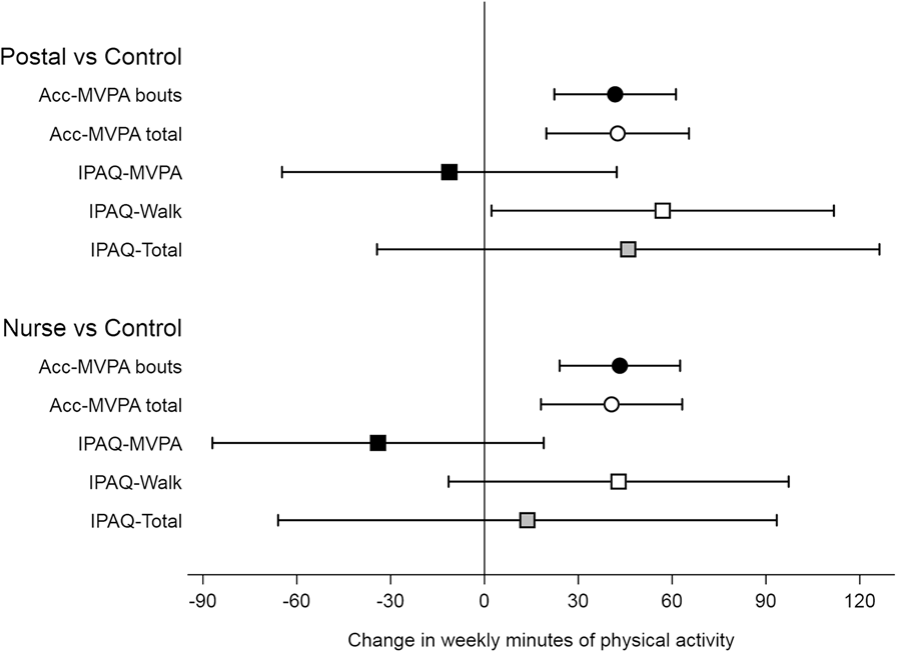
Treatment effects and 95% confidence intervals for change in minutes of physical activity measured by accelerometry, IPAQ-MVPA, IPAQ-Walk and IPAQ-Total

Both intervention groups showed statistically significant increases in accelerometer-MVPA, both in bouts and total, compared with controls. Increases in accelerometer-MVPA bouts: postal group 42 min/week (95% CI 22 to 61), nurse group 43 (95% CI 24 to 63) (Table 2 and Fig. 1a); increases for total accelerometry MVPA were almost identical to accelerometer-MVPA in bouts but with wider confidence intervals (Table 2 and Fig. 1). Repeating the analysis using the IPAQ outcomes, IPAQ-Walk showed positive increases, similar in magnitude to accelerometer-MVPA in the nurse group, but with wider confidence intervals indicating less precision: postal group 57 min (95% CI 2 to 112), nurse group 43 (95% CI -11 to 97). IPAQ-MVPA showed non-significant decreases and IPAQ-Total showed non-significant increases. The distribution of residuals from the regression models were normally distributed for MVPA in bouts [27] and IPAQ outcomes (data not shown).

**Table 2:** Physical activity outcomes (total weekly minutes) at baseline and 12 months for accelerometry and IPAQ

	Group summary data												Treatment effects			
	Control group (*n* = 231)				Postal group (*n* = 207)				Nurse group (*n* = 217)				Postal vs Control		Nurse vs Control	
	Baseline		12 months		Baseline		12 months		Baseline		12 months		Effect	*p*-value	Effect	*p*-value
	Mean	(sd)	Mean	(sd)	Mean	(sd)	Mean	(sd)	Mean	(sd)	Mean	(sd)	(95% CI)		(95% CI)	
Accelerometry outcomes																
Daily step count	7572	(2738)	7402	(2724)	7691	(2560)	8233	(3076)	7487	(2738)	8146	(3224)	804 (426, 1181)	< 0.001	837 (463, 1211)	< 0.001
MVPA in ≥10 min bouts (weekly minutes)	95	(103)	97	(101)	107	(95)	144	(128)	107	(114)	146	(149)	42 (22, 61)	< 0.001	43 (24, 63)	< 0.001
Total MVPA (weekly minutes)	319	(155)	330	(160)	329	(143)	377	(173)	317	(157)	367	(189)	43 (20, 65)	< 0.001	41 (18, 63)	< 0.001
IPAQ outcomes																
IPAQ-MVPA (weekly minutes)	188	(300)	222	(343)	171	(285)	200	(288)	165	(249)	180	(300)	-11 (−65, 42)	0.68	−34 (−87, 19)	0.21
IPAQ-Walk (weekly minutes)	336	(332)	356	(335)	331	(336)	398	(332)	286	(262)	365	(309)	57 (2, 112)	0.04	43 (−11, 97)	0.12
IPAQ-Total (weekly minutes)	525	(494)	578	(520)	502	(481)	598	(479)	450	(365)	545	(456)	46 (−34, 126)	0.26	14 (−66, 93)	0.74

### ii) Comparison of estimated treatment effects using the binary variable “active”

Similar patterns were found for the binary variable “active” for the different outcomes. Odds ratios (ORs) for being “active” at 12 months (achieving ≥150 weekly minutes of MVPA in ≥10 min bouts) conditional on baseline “active” status were statistically significant for accelerometry-MVPA: postal group 3.7 (95% CI 1.8 to 7.5) and nurse group 2.9 (95% CI 1.5 to 5.7) (Table 3). IPAQ-Walk showed statistically significant OR for the postal group, 2.1 (95% CI 1.2 to 4.0) and borderline for the nurse group, 1.7 (95% CI 1.0 to 3.0). Results were inconclusive for IPAQ-MVPA and IPAQ-Total had increased ORs for both intervention groups, but only statistically significant for the nurse group. ORs for the two GPPAQ outcomes were close to 1.0 suggesting that GPPAQ was unable to identify changes in the proportion classified as “active” (Table 3). Sensitivity analyses using ≥180 min of the accelerometer and IPAQ outcomes gave similar results.

**Table 3:** Physical activity outcomes (“active”) at baseline and 12 months for accelerometry, IPAQ and GPPAQ

	Group summary data												Treatment effects			
	Control group (*n* = 228)				Postal group (*n* = 205)				Nurse group (*n* = 213)				Postal vs Control		Nurse vs Control	
	Baseline		12 months		Baseline		12 months		Baseline		12 months		OR	*p*-value	OR	*p*-value
	N	(%)	N	(%)	N	(%)	N	(%)	N	(%)	N	(%)	(95% CI)		(95% CI)	
Accelerometry																
MVPA bouts: 150 mins	54	(24%)	47	(21%)	53	(26%)	83	(40%)	55	(26%)	79	(37%)	3.7 (1.8, 7.5)	< 0.001	2.9 (1.5, 5.7)	0.002
MVPA total: 150 mins	199	(87%)	200	(88%)	180	(88%)	185	(90%)	183	(86%)	193	(91%)	1.7 (0.7, 3.9)	0.24	1.7 (0.7, 3.8)	0.24
IPAQ																
IPAQ-MVPA: 150 mins	81	(36%)	90	(39%)	68	(33%)	89	(43%)	77	(36%)	76	(36%)	1.4 (0.6, 3.3)	0.38	0.6 (0.3, 1.4)	0.27
IPAQ-Walk: 150 mins	148	(65%)	156	(68%)	136	(66%)	161	(79%)	137	(64%)	162	(76%)	2.1 (1.2, 4.0)	0.01	1.7 (1.0, 3.0)	0.05
IPAQ-Total: 150 mins	190	(83%)	189	(83%)	177	(86%)	182	(89%)	178	(84%)	194	(91%)	1.8 (0.9, 3.5)	0.07	2.3 (1.1, 4.6)	0.02
GPPAQ																
PA Index: Active ≥180 mins PA per week	28	(12%)	37	(16%)	31	(15%)	37	(18%)	24	(11%)	28	(13%)	1.1 (0.6, 2.1)	0.83	0.8 (0.4, 1.6)	0.50
PA Index incl walking: Active ≥180 mins PA per week including walking at brisk/fast pace	66	(29%)	77	(34%)	62	(30%)	74	(36%)	59	(28%)	70	(33%)	1.1 (0.7, 1.9)	0.66	1.0 (0.6, 1.8)	0.89

## Discussion

The PACE-UP study was a walking intervention designed to increase individuals’ PA through a 3-month programme, in particular MVPA in ≥10 min bouts in line with current UK, WHO and US PA guidelines [31–33]. We found statistically significant increases between baseline and 12 months in accelerometer measured MVPA in ≥10 min bouts for both intervention groups compared with control. IPAQ-Walk showed a significant increase in the postal group and a non-significant increase in the nurse group compared with control, but with less precision than with accelerometry. IPAQ-MVPA showed non-significant decreases and IPAQ-Total non-significant increases in intervention groups compared with controls. When considering the proportion of “active” individuals, only accelerometry showed statistically significant increases for both intervention groups versus controls. IPAQ-Walk and IPAQ-Total showed statistically significant increases for one intervention group compared with controls (postal for IPAQ-Walk and nurse for IPAQ-Total), but borderline effects for the other intervention group compared with controls. Neither IPAQ-MVPA nor GPPAQ identified any change in the proportions categorised as “active” in intervention versus control groups. Therefore, in terms of overall construct validity for assessing change in walking in a walking intervention study, accelerometry has the greatest validity, followed by IPAQ-Walk. The other measures have considerable disadvantages: IPAQ-MVPA and GPPAQ have very poor construct validity; IPAQ-Total is measured with substantial imprecision and is unsuitable for assessing a walking intervention as it includes IPAQ-MVPA.

Our study had several strengths. It was based on a large population-based sample of adults from seven south-west London (UK) general practices (family practices), predominantly classified as inactive at baseline. Accelerometry is an objective PA measure and measures walking accurately. We used standard cut-points to define the different intensities of accelerometry activity and were thus able to identify those bouts of walking which can be classified as MVPA. The main PACE-UP analysis [27] showed that the increase in weekly steps in intervention groups relative to control group was equivalent to the increase in weekly minutes of MVPA and this was all in ≥10 min bouts, thus demonstrating the effectiveness of the PACE-UP walking intervention. The two self-completed questionnaires, IPAQ and GPPAQ, are standard questionnaires used to assess PA, and were completed for the same 7 days as for accelerometry, thus providing directly comparable estimates of effect. The study achieved 93% accelerometry follow-up at 12 months, > 90% of these with ≥5 days wear-time. Total weekly minutes of MVPA and total weekly minutes of walking (not including MVPA) were easy to extract from IPAQ and provided a direct comparison with minutes of accelerometer-MVPA. The increases in IPAQ-Walk minutes are similar to those for accelerometer-MVPA suggesting that IPAQ can identify changes in walking minutes, although the wider confidence intervals show the loss of precision from using IPAQ. At baseline, average IPAQ-Walk minutes were similar to average total accelerometer-MVPA minutes rather than accelerometer-MVPA in ≥10 min bouts. This is perhaps unsurprising, as the IPAQ walking questions ask for number of days walking and duration on each day, and people may find it easier to report walking minutes as a rounded number e.g. 30 or 45 min per day and which may include relative short walks of < 10 min. GPPAQ is commonly used in UK general practice to assess an individual’s PA. However, it can underestimate PA amongst those not working or those whose main PA is walking, and this study provided a further opportunity to evaluate our modified GPPAQ–Walk index [29]. We were also able to estimate how well GPPAQ could identify individuals moving from “not active” to “active” (assumed to be achieving PA guidelines). Finally, our method of analysis, regressing outcome at 12 months on baseline values focusses on individual changes in activity while allowing for regression to the mean. Cross-sectionally, the distributions of accelerometer-MVPA and IPAQ measures are highly skewed leading many to present medians and interquartile ranges of activity at different time points. However, change in activity is usually symmetric and reasonably normally distributed, which our approach exploits. We were thus able to present mean changes in activity and associated confidence intervals for both accelerometry and questionnaire measures, thus allowing for a more informative comparison.

The study also had some important limitations. All of the PA measures (accelerometry, IPAQ and GPPAQ) only measured PA levels for 7 days and it may be that participants were more likely to be active or report being active in the week that their PA was being assessed, rather than at other times. However, any such tendency would potentially affect all of the PA measures and would be true for control participants as well as for those in the intervention group. IPAQ is difficult to complete and thus unreliable if an individual’s PA varies by day across the week. Although we had high return rates at baseline and 12 months for the IPAQ and GPPAQ, 97% and 92% respectively, each IPAQ outcome at baseline and 12 months had 20–25% missing or incomplete answers. Participants’ comments on the questionnaires described their confusion over how to interpret and answer the questions and many questions were left blank. This reduced our sample size to 655 for comparisons with accelerometry although this is still large compared with other studies [21–23]. The proportions of missing data were similar across the three groups, but those with missing IPAQ data had lower mean accelerometry-MVPA at baseline and 12 months than those with complete data. The accelerometry effect sizes reported here (42–43 min) are also larger than for the full cohort (33–35 min) [27]. The limited options on GPPAQ for duration of PA, led to using ≥3 h (180 min) for GPPAQ “active” whereas the PA guidelines are ≥150 min. However, ORs from sensitivity analyses using ≥180 min for accelerometry and IPAQ outcomes were similar to those using ≥150 min. Although neither of our methods of measuring PA are considered a gold-standard, accelerometry has the advantage of providing an objective time-stamped record of PA that does not rely on recall. It has been validated as a measure of activity energy expenditure using doubly labelled water [15] and we used standard cut-points in counts per minute to define MVPA. [16] Our findings that accelerometer-MVPA and IPAQ-Walk provide similar estimates of change clearly support results from the PACE-UP intervention which is aimed at increasing walking, but it is unknown if these findings would be generalisable to other PA interventions.

Participants in the postal and nurse intervention groups were encouraged to increase their MVPA through walking and the nurse group in particular were taught to recognise and classify different PA intensities – vigorous, moderate, light, and sedentary. Thus they may have been more likely to accurately report their PA on IPAQ at follow-up i.e. with less over-estimation of their PA levels, which could explain the non-significant decreases in the treatment groups for IPAQ-MVPA from the modelling.

### Comparison with other studies

Our baseline data agree with other studies that individuals tend to over-estimate their PA on self-report questionnaires compared with objective accelerometry, both time spent being physically active [6] and proportions achieving PA guidelines [7]. Studies which have found better correspondence between IPAQ and accelerometry cross-sectionally [34] have used total accelerometer MVPA rather than MVPA in ≥10 min bouts and a similar pattern is seen in our data where baseline total accelerometer-MVPA minutes are similar to IPAQ-Walk minutes. However, IPAQ questions ask about vigorous and moderate PA in ≥10 min bouts and UK, WHO and US PA guidelines are based on ≥150 min of MVPA per week in ≥10 min bouts. In our trial, whilst total accelerometry MVPA was much higher than accelerometry MVPA in ≥10 min bouts, changes in both measures were almost identical.

To our knowledge, this is the largest population-based trial to make direct comparisons of accelerometry and self-report questionnaires to assess an individual’s change in minutes of PA after an intervention. There are limitations with all five studies we identified [21–25] which have attempted to compare longitudinal changes in PA measured using IPAQ compared with objective measures. Three studies recruited less than 100 subjects [21–23]. One study was observational [21], one had no control group [22] and one was a weight loss intervention rather than PA intervention [24]. One study was comparing IPAQ with pedometer steps [23] and another with accelerometer counts [25] making direct comparison of minutes of physical activity between IPAQ and accelerometry difficult. Whilst our study compares measures using different constructs, we were able to compare time spent in MVPA and time spent walking, both in minutes per week. Three studies present distribution of PA measures at baseline and follow-up, but provided no estimate of the distribution of change [21, 24, 25]. Our findings do agree with two of the small studies. Nicaise et al. [22] followed up one group of women, but with no control group, and found median changes in IPAQ Walking minutes were similar to median changes in accelerometer MVPA minutes. Baker et al. [23] compared IPAQ PA minutes with pedometer steps, and argue that the increase in step counts in the intervention group was comparable to the increase in leisure time walking reported on IPAQ, although they report mean differences for pedometer steps and median differences for IPAQ data.

GPPAQ is used in UK primary care to help identify those not achieving PA guidelines during UK NHS Health Checks [12]. GPPAQ guidance recommends repeating it annually on those at increased cardiovascular risk [11], but our study suggests that it is poor at identifying those individuals who have increased their PA to current guideline levels. In addition, the binary nature of this outcome fails to recognise modest, but important, increases in PA made by inactive individuals. We have also confirmed our previous findings [29] that, compared with objective accelerometry, GPPAQ underestimates the proportion of “active” individuals and our modified index GPPAQ-Walk classifies slightly more as “active”.

## Conclusions

We have demonstrated that neither GPPAQ nor IPAQ-MVPA provide a valid estimate of change in a walking intervention trial compared with accelerometry measures. Moreover, we have shown that although IPAQ-Walk produces an estimate of change comparable with that from accelerometry MVPA in ≥10 min bouts, the IPAQ-Walk estimate had considerably less precision. Missing data were also an issue with the self-report IPAQ. This has implications for future trials. Studies may need to use IPAQ to assess changes in walking if they are not able to use accelerometry. If this is the case, they should focus particularly on the walking questions and will need to be larger to be adequately powered, although they will still lack information on intensity of any changes that occur. In conclusion, accelerometry is preferred to self-report measures in assessing the effects of a walking intervention, as it avoids recall bias and improves precision.

## Abbreviations

95% CI: 95% Confidence Interval
GPPAQ: General Practice Physical Activity Questionnaire
IPAQ: International Physical Activity Questionnaire
MVPA: Moderate-to-vigorous physical activity
NHS: UK National Health Service
OR: Odds ratio
PA: Physical activity
sd: Standard deviation
